# Chemical compositions and biological activities of *Serevenia buxifolia* essential oil leaves cultivated in Vietnam (Thua Thien Hue)

**DOI:** 10.1002/fsn3.3395

**Published:** 2023-05-10

**Authors:** Anh Vo Bui, Thanh Vy Pham, Kim Ngan Nguyen, Nhat Tan Nguyen, Khanh Duy Huynh, Van‐Son Dang, Tomas Ruml, Dieu‐Hien Truong

**Affiliations:** ^1^ Faculty of Applied Sciences Ton Duc Thang University Ho Chi Minh City Vietnam; ^2^ Institute of Tropical Biology Vietnam Academy Science and Technology Ho Chi Minh City Vietnam; ^3^ Faculty of Food and Biochemical Technology University of Chemistry and Technology Prague Czech Republic

**Keywords:** biological activity, essential oil, GC/MS, *Serevenia buxifolia*

## Abstract

*Serevenia buxifolia* is an evergreen citrus plant and has attracted considerable attention due to its bioactive components and biological activities. In the present study, the essential oil (EO) from *S. buxifolia* cultivated in Vietnam was demonstrated to exhibit the in vitro antioxidant, thrombolytic, anti‐hemolysis, anti‐inflammatory, and antidiabetic activities. Briefly, the gas chromatography coupled to mass spectrometry analysis revealed that the leaf EO of *S. buxifolia* was composed of 33 components, with the main constituents being β‐carypphyllene (32.5%), and elixene (9.8%). The extracted oil possessed a fairly high free radical scavenging activity against 2, 2‐diphenyl‐1‐picrylhydrazyl (DPPH), with an IC_50_ value of 190.7 μg/mL compared with positive control, α‐tocopherol, IC_50_ value of 42.6 μg/mL. The EO also exhibited thrombolytic activity: the percentage of inhibition was found to be 70.75% at 100 μL, in comparison with 87.2% for the positive control, streptokinase. For hemolytic activity, the percentage of inhibition of the EO was from 27.4% to 59.6% at concentrations from 10 to 100 μg/mL, respectively. The results of in vitro anti‐inflammatory activity indicated that the EO of *S. buxifolia* leaves effectively protects the heat‐induced denaturation, with an IC_50_ value of 40.25 μg/mL. The EO also exhibited antidiabetic potential, with IC_50_ values of 87.8 and 134.9 μg/mL against α‐amylase and α‐glucosidase, respectively. It is noteworthy that the potent biological activities of the obtained *S. buxifolia* oil increased in a dose‐dependent manner. The results achieved show that the EO of *S. buxifolia* leaves can be a potential source for oxidative stress, inflammatory, and diabetic management.

## Graphical abstract

Essential oil of plant leaves. Biological activity of essential oil. Chemical compositions of essential oil.

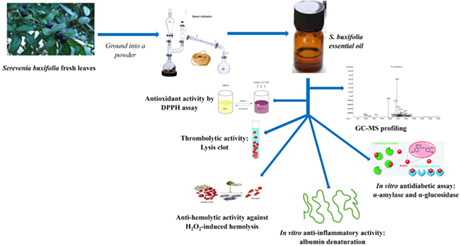

## 1 INTRODUCTION

The increased resistance to drugs, especially antibiotics, has intensified due to the drug overuse. Hence, there is growing interest in their replacement with natural products (Hayet et al., 2017). One such possibility may be the use of herbal and botanical products like essential oils (EOs) and plant extracts exhibiting desired biological activities (Brah et al., 2023; Li et al., 2022; Manzur et al., 2023; Shen et al., 2022). For example, the potent antibacterial (Cazella et al., 2019; Ghavam et al., 2020; Man et al., 2019), antifungal (Gakuubi et al., 2017; Nazzaro et al., 2017), insecticidal (Kweka et al., 2011; Owolabi et al., 2014), acaricidal (Djebir et al., 2019), cytotoxic (Khang et al., 2020; Russo et al., 2015), antioxidant (Cardoso‐Ugarte & Sosa‐Morales, 2021; Torres‐Martínez et al., 2018), and antidiabetic (Siahbalaei et al., 2020) properties of EOs have been investigated. EOs (also called volatile or ethereal oils) are the aromatic oily liquids liberated from plant material (e.g., seeds, leaves, buds, flower, barks, wood, fruits, and roots; Burt, 2004).

Among the 20 species of genus *Serevenia* (Rutaceae family), *Serevenia buxifolia* (*Atalantia buxifolia*) is an evergreen citrus plant which has attracted considerable attention due to its bioactive components and biological activities (Le et al., 2020; Liang et al., 2020; Rao et al., 2021; Satyal et al., 2019). Studies have documented that *S. buxifolia* plants are a promising source of alkaloids, terpenoids, phenolics, and other compounds (Chang et al., 2018; Truong et al., 2021; Wu et al., 2001). For example, Wu et al. (2001) identified two tetraterpenoids (7‐isovaleroylcycloseverinolide and 7‐isovaleroylcycloepiatalantin) in the ethanol extract of the root barks of *S. buxifolia*. Several sesquiterpenes were also isolated from the root bark of *S. buxifolia*, such as α‐santalene, α‐santalen‐11‐one, dihydro‐α‐santalen‐12‐one, and 12,13‐ epoxy‐α‐santalene.

In Vietnam, although *S. buxifolia* is widely used as a traditional medicine for cough, snakebites, malaria, chronic rheumatism, influenza, sore throat, and pain (Satyal et al., 2019; Truong et al., 2019, 2021), studies on the chemical compositions and pharmaceutical effects of this plant are still limited. In previous studies, we have detected the presence of phenolics, flavonoids, alkaloids, and terpenoids in the crude and fraction extracts of *S. buxifolia* branches and stems. The potent in vitro antioxidant and anti‐inflammatory action of such extracts have also been investigated. Considering the EO of *S. buxifolia*, Satyal et al. (2019) have recently identified the oil compositions of *S. buxifolia* leaves cultivated in Da Nang city (in the middle of Vietnam), with sabinene, β‐caryophyllene, bicyclogermacrene, germacrene D, (E)‐nerolidol, globulol, and linalool being the main components. The EO also showed larvicidal activity and repellent activity against *Triatoma rubrofasciata*, *Aedes aegypti*, and *A. albopictus*.

In order to exploit local species and to continue the previously successful studies on *S. buxifolia* extracts, we aimed in this work on the investigation of biological activities of EO from *S. buxifolia* leaves. No scientific studies have been conducted regarding the in vitro antioxidant, thrombolytic, anti‐hemolytic, anti‐inflammatory, and antidiabetic activities of *S. buxifolia* leaf oil. Therefore, the present study was undertaken to evaluate the in vitro antioxidant, thrombolytic, anti‐hemolytic, anti‐inflammatory, and antidiabetic activities of the essential oil of *S. buxifolia* leaves collected from Thua Thien Hue, Vietnam. Besides, the phytochemical composition of the leaf oil was also identified by using gas chromatography coupled to mass spectrometry (GC/MS).

## 2 MATERIALS AND METHODS

### 2.1 Chemical reagents and solvents

Phosphate‐buffered saline (PBS), 4‐Nitrophenyl β‐D‐glucopyranoside (pNPG), 3,5‐dinitro salicylic acid (DNS), caryophyllene, bovine serum albumin (BSA), α‐tocopherol, α‐amylase, α‐glucosidae, streptokinase, aspirin, 2, 2‐diphenyl‐1‐picrylhydrazyl (DPPH), and acarbose were purchased from Sigma‐Aldrich, Singapore. All other reagents and solvents were of analytical grade.

### 2.2 Plant materials and extraction procedure

The fresh leaves were collected from *S. buxifolia* in the Phu Loc district, Thua Thien Hue province, Vietnam in the summer season (from August to September, 2020). The plant was taxonomically identified by identified by Dr. Son V. Dang, Curator of the VNM Herbarium, Institute of Tropical Biology, Vietnam. The leaves were lightly washed with tap water to remove debris and then ground into a powder using a mill (Jehmlich) and kept at 4–6°C until further use.

The leaf EO of *S. buxifolia* was extracted by steam distillation for 5 h, as described by Božović et al. (2017), with some minor modifications. The mixture of EO and water was then dried over anhydrous sodium sulfate and stored at 4°C until required in the bioassay. The extract yields (EY) were calculated as the ratio of the mass of EO to the mass of the starting plant materials and were expressed as a percentage (w/w).

### 2.3 Chemical analysis of essential oil

The EO of Serevenia buxifolia leaves was analyzed by GC/MS, as described by Liu et al. (2014).

The components of the EO were separated by GC and identified by MS using an Agilent GC 6890 equipped with an HP‐5MS (5%‐Phenylmethylpolysiloxane) fused silica column (30 m × 0.25 mm × 0.25 μm) interfaced with a quadrupole detector (Model 5973). The GC settings were as follows: the initial oven temperature was held at 50°C for 2 min and ramped at 2°C/min to 80°C, then ramped at 5°C/min to 150°C, where it was held for 5 min, before being ramped at 10°C/min to 200°C and then at 20°C/min to 300°C, where it was held for 10 min. The sample (1 μL diluted 1:100 in hexane) was injected, with a split ratio of 1:50. The carrier gas was helium, at a flow rate of 1 mL/min. Spectra were obtained over the scan range from 20 to 500 m/z at 2 scans/s.

#### 2.3.1 Qualitative analysis

Most components were identified based on the retention indices (RI) determined with reference to a homologous series of n‐alkanes, under identical experimental conditions, co‐injection with authentic samples or known EO compositions, MA library search (NIST 08 and Wiley 9th Version), and by comparing with MS literature data of Adams (2007).

#### 2.3.2 Quantitative analysis

The percentage compositions of the oil depending on whether there was computed by the normalization method from the GC peak areas, assuming an identical mass response for all components. In addition, the content of the main oil component was also calculated according to the standard curve of the commercial compound.

### 2.4 Assessment of biological activities

#### 2.4.1 Antioxidant activity: DPPH radical scavenging activity

DPPH assays were applied to determine the antioxidant activity of the EOs from *S. buxifolia* leaves, as described by Ndoye Foe et al. (2016), with some slight modifications. The test samples were prepared in ethanol, and 100 and 500 μL of each sample (100, 150, 200, and 250 μg/mL of EO) were added to 1 mL of freshly prepared DPPH solution (0.004%) in pure ethanol. α‐tocopherol was used as a positive control, and ethanol was used as a negative control. The content of each preparation was mixed and incubated at 37°C in the dark for 30 min. The absorbance was measured at 517 nm in a V‐730 spectrophotometer (Jasco). The radical scavenging activity (RSA) was calculated as a percentage of DPPH radical scavenging, using the equation below:

$$\%\mathrm{RSA}=\left[\left(A_{\text{blank}}–A_{\text{sample}}\right)/A_{\text{blank}}\right]\;x\;100$$

where *A*_blank_ is the absorbance of the control (containing all reagents except the test sample) and *A*_sample_ is the absorbance of the tested EO solution.

The antioxidant potential of the EO or standard was expressed as an IC_50_ value (μg/mL), defined as the concentration of the tested sample required to cause a 50% decrease in initial DPPH concentration.

#### 2.4.2 Thrombolytic activity

The in vitro thrombolytic activity of the EO was established according to the method of Prasad et al. (2006), with some minor modifications. A 5 mL sample of venous blood was drawn from healthy human volunteers (*n* = 5), without recent history of anticoagulant and contraceptive therapy (at least 7–10 days duration). Then, the blood samples were immediately transferred in different pre‐weighed sterile Eppendorf tubes (500 μL/tube; m_0_) and incubated for 45 min at 37°C. Next, the serum was completely removed after clot formation (working carefully to avoid disturbing the formed clot). Later, each tube including clot was weighed again (m_1_) to determine the clot weight according to the following equation:

weight of clot=weight of clot filled tube−weight of empty tube

Each Eppendorf tube with clot was properly labeled, and 100 μL of different dilutions of EO in dimethyl sulfoxide (DMSO; 10, 20, 50, and 100 μg/mL) was added to the tubes. DMSO was considered as a negative thrombolytic control, while the thrombolytic drug streptokinase (30,000 IU) was used as a positive standard stock (10, 20, 50, and 100 μg/mL). After incubation for 90 min at 37°C, the fluids obtained in all the tubes were removed, and tubes were again weighed to observe the variation in weight after clot disruption. The difference obtained from the weight taken before and after clot lysis was expressed as a percentage of clot lysis:

$$\%\text{of clot lysis}=\left[\left(\text{Weight of the clot before lysis}-\text{Weight of the clot after lysis}\right)/\text{Weight of the clot before lysis}\right]\;x\;100$$

#### 2.4.3 Anti‐hemolytic activity against H_2_O_2_ _−_induced hemolysis

The anti‐hemolytic potential of *S. buxifolia* leaf oil was measured according to the method of Vinodhini and Kalaiselvi (2021), with slight modifications. First of all, 5 mL samples of venous blood from healthy human volunteers were collected in EDTA tubes (10%) and centrifuged at 1008 *g* (EPA 200) for 10 min. After centrifugation, the plasma was discarded from the tubes, and the settled red blood cells (RBCs) were washed three times with an equal volume of normal saline (0.9% NaCl). The RBCs were then diluted with PBS (pH 7.4) to give a 4% (v/v) suspension, and 0.4 mL of EO diluted in PBS to different concentrations (10, 20, 50, and 100 μg/mL) was then added to 2.0 mL of the RBC suspension, and the volume was made up to 5.0 mL with saline. This mixture was later incubated for 5 min at room temperature before adding 0.5 mL of H_2_O_2_ (PBS) solution in buffered saline to induce oxidative degradation of the membrane lipids. All tubes were incubated for 1 h at room temperature, and the reaction mixture was then centrifuged at 252 *g* (Hematokrit 210) for 10 min. Finally, the hemolysis inhibitory ability was measured spectrophotometrically at 540 nm. The relative hemolysis of the EO was assessed compared to the hemolysis in the H_2_O_2_‐treated sample (negative control), which was set as 100%. PBS was used as a positive control. The percent inhibition of hemolysis was calculated as follows:

$$\%I=\left[\left({\mathrm{Abs}}_{\text{control}}–{\mathrm{Abs}}_{\text{sample}}\right)/{\mathrm{Abs}}_{\text{control}}\right]\;x\;100$$

The concentrations of extracts causing 50% inhibition of enzyme activity (IC_50_) were determined graphically. All products were evaluated with the comparison of their IC_50_ values estimated from the dose–response curves.

#### 2.4.4 In vitro anti‐inflammatory activity

The in vitro anti‐inflammatory ability of EO from *S. buxifolia* leaves was determined by the inhibition of albumin denaturation, as described by (Vinodhini & Kalaiselvi, 2021), with a few modifications. The reaction mixture consisted of 1 mL of EO at different concentrations (10, 20, 50, and 100 μg/mL diluted in DMSO) and 1 mL of 1% aqueous solution of BSA. The pH of the mixture was adjusted to 6.3 using 1 N HCl. Next, the samples were incubated at 37°C for 20 min before being heated to 57°C for 20 min. The sample was cooled and the turbidity was measured spectrophotometrically at 660 nm. The inhibitory percentage of protein denaturation was calculated as follows:

$$\%I=\left[\left({\mathrm{Abs}}_{\text{control}}–{\mathrm{Abs}}_{\text{sample}}\right)/{\mathrm{Abs}}_{\text{control}}\right]\;x\;100$$

#### 2.4.5 Determination of in vitro antidiabetic activity

The potential antidiabetic activity of the EO of *S. buxifolia* leaves was evaluated based on the percentage of α‐amylase and α‐glucosidase inhibition.

#### 2.4.6 α‐Amylase inhibitory method

The α‐amylase inhibition assay of the EO of *S. buxifolia* leaves was adapted from Jaradat et al. (2020), with slight modifications. A working solution (1 mg/mL) of EO was prepared by dissolving 25 mg in a small amount of DMSO; later, a buffer solution was added up to 25 mL. This solution was then diluted with DMSO to obtain various dilutions (50, 100, 150, and 200 μg/mL). The α‐amylase enzyme working solution (2 U/mL) was produced by dissolving 12.5 mg of α‐amylase enzyme powder in a small amount of DMSO, and the buffer solution was made up to 100 mL. Corn starch solution was obtained by dissolving 1 g of starch in 100 mL of distilled water. The reaction mixture containing 200 μL of each EO stock solution and 200 μL of α‐amylase stock solution was incubated at 30°C for 10 min. Next, 200 μL of corn starch solution was added, before continuing the incubation at 30°C for 3 min. The enzyme reaction was terminated by adding 200 μL of DNS and heating at 85–90°C for 10 min. The solution was then cooled and 5 mL of distilled water was added. Acarbose was used as the reference standard (positive control), while DMSO (200 μL) was used as the blank solution. The α‐amylase inhibitory potential was measured spectrophotometrically at 540 nm and estimated using the following formula:

$$\%I=\left[\left({\mathrm{Abs}}_{\text{control}}–{\mathrm{Abs}}_{\text{test}}\right)/{\mathrm{Abs}}_{\text{control}}\right]\;x\;100\%$$

where %*I* is the percentage of α‐amylase inhibition Ali et al. (2006). The results were expressed in terms of IC_50_ values, indicating the concentration of EO required to cause 50% enzyme inhibition.

#### 2.4.7 α‐Glucosidase inhibitory method

The α‐glucosidase assay was performed according to Siahbalaei et al. (2020), with some modifications. The reaction mixture containing 300 μL of each concentration of EO (50, 100, 150, and 200 μg/mL diluted in DMSO from stock solution (1 mg/mL)) and 200 μL of α‐glucosidase (0.3 U/mL) was incubated at 37°C for 15 min in the dark. Next, 100 μL of pNPG (10 mM) was added before incubating at 37°C for 30 min. Later, 300 μL of Na_2_CO_3_ (100 mM) was added, and the absorbance was recorded at 405 nm. Acarbose was used as the reference standard, while DMSO was used as the blank control. The percentage of α‐glucosidase inhibitory potential of the EO was calculated in the same manner as in the α‐amylase assay. Acarbose, a prescribed drug for α‐glucosidase inhibition, was also used as a control. The blank solution was prepared by replacing the EO with 200 μL of DMSO.

### 2.5 Statistical analysis

All experiments were performed in triplicates, and values were represented as the mean ± standard deviation (SD). Analysis of variance (ANOVA; Minitab 16 software) and Tukey's multiple comparison test were used to compare the variation between assays; *p* values <.05 were considered significant.

## 3 RESULTS AND DISCUSSION

### 3.1 Essential oil analysis

Essential oil obtained by steam distillation of the *S. buxifolia* leaves was extracted with a yield of 0.31 ± 0.01 (w/w). The density of the concentrated oil was 0.87 g/mL. The isolated EO was a yellowish clear liquid with strong aromatic fragrances (characteristic of Rutaceae EOs) and presented a high solubility in ethanol and DMSO.

**FIGURE 1:**
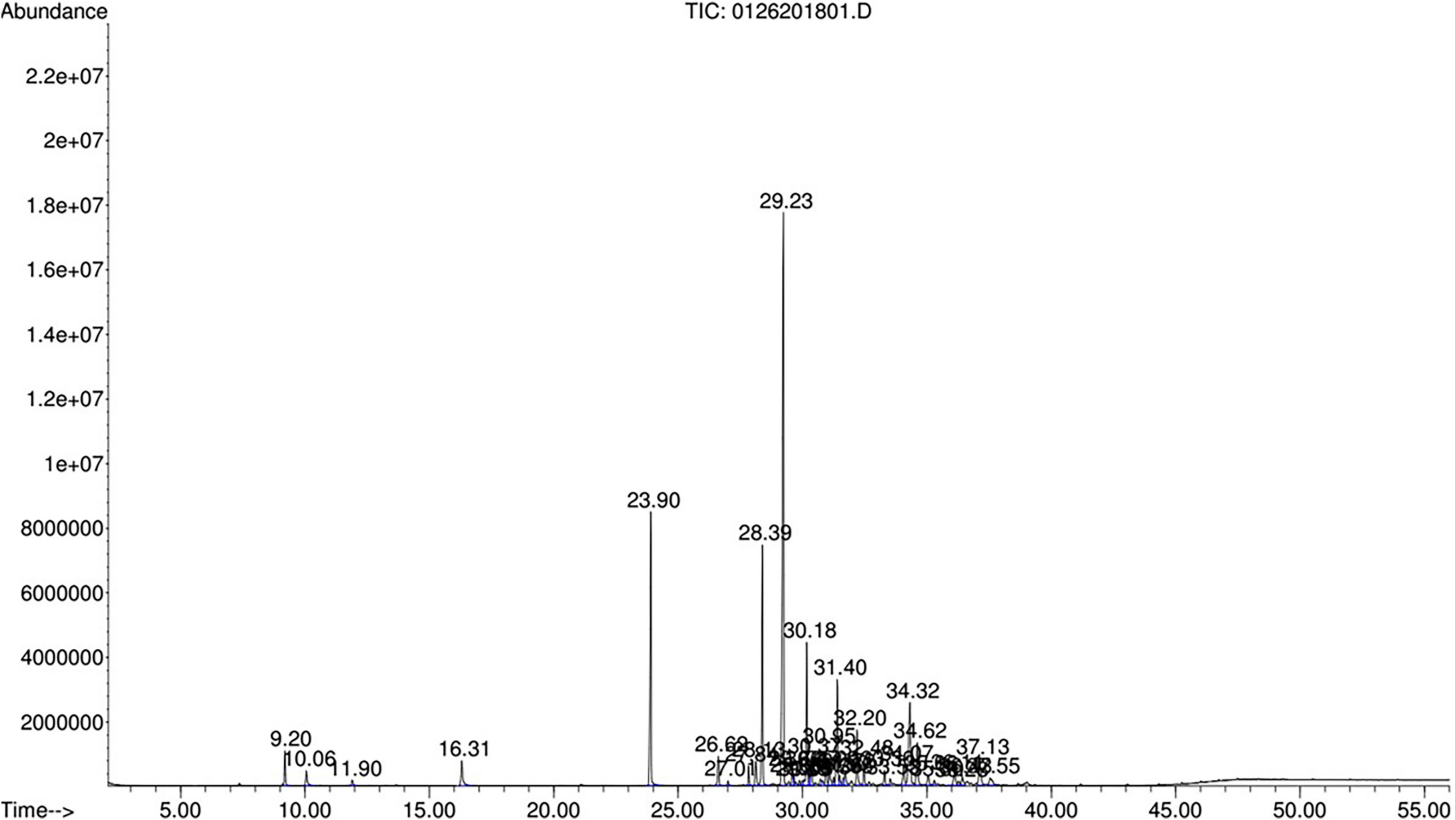
GC/MS chromatogram of essential oil derived from *Serevenia buxifolia* leaves cultivated in Thua Thien Hue, Vietnam.

The results of GC–MS analysis showed that the EO of *S. buxifolia* leaves exhibited 33 identified components by comparison of their retention times and the mass spectra of each GC composition with those of the standards, accounting for 97.5% of the total oil (Table 1 & Figure 1). The main components of the EO were β‐caryophyllene (32.5%), elixene (9.8%), germacrene D (6.9%), β‐farnesene (6.6%), δ‐cadinene (6.4%), caryophyllene oxide (4.9%), and β‐elemene (4.6%), followed by D‐limonene (3.6%) and linalyl acetate (3.2%). Monoterpenoids represented nine of the 33 components, corresponding to 10.5% of the whole oil, whereas 21 of the 33 constituents were sesquiterpenoids (83.2% of the crude EO). In addition, three of the 33 components were fatty alcohol esters (aliphatic acyclic compounds), corresponding to 2.9% of the crude oil. This chemical profile was close to that found in the same *Severinia* species cultivated either in Da Nang, Vietnam by Satyal et al. (2019) or Dakrong District, Quang Tri Province, Vietnam by Le et al. (2020). There was a slight difference compared to the study of Satyal et al. (2019), where the major components of EO of *S. monophylla* cultivated in Da Nang city, Vietnam were sabinene, β‐caryophyllene, bicyclogermacrene, germacrene D, (E)‐nerolidol, globulol, and linalool. The major EO of *A. sessiflora* (*S. buxifolia*) leaves located in Quang Tri Province, Vietnam was found to be linalool (16.21%), E‐β‐caryophyllene (11.01%), ledene (8.59%), α‐humulene (8.02%) and L‐α‐terpineol (6.99%; Le et al., 2020). The differences in chemical compositions of the EOs in the present study and in previous studies may probably be due to different genetic and environmental factors, the nutritional status of the plant, and/or other factors (Yang et al., 2015). Evidences indicate that the place of origin, climatic conditions, and seasons can be key factors influencing the chemical compositions of EOs (Adlard, 2010; Martínez‐Razo et al., 2021). Besides, some components can be resulted from the decomposing process of the precursor compositions during distillation techniques (Le et al., 2020; Martínez‐Razo et al., 2021). Many studies have indicated that β‐carypphyllene marks the characteristics of the Rutaceae EO (Dai et al., 2012; Das & Swamy, 2013; Padalia et al., 2012; Pang et al., 2021; Vinturelle et al., 2017). For example, Das and Swamy (2013) presented that caryophyllene, decanal, and D‐limonene were large amounts in the *Atalantia wightii* EO, whereas T‐cadinol, caryophyllene, and caryophyllene oxide were found to be the main components in the EO of *Atalantia racemosa*.

**TABLE 1:** Chemical constituents of essential oil derived from *Serevenia buxifolia* leaves cultivated in Thua Thien Hue, Vietnam.

Peak no.	Compound	RI^a^	RI^b^	Percent composition
Monoterpene hydrocarbons				
1	β‐myrcene	989	988	0.2
2	α‐phellandrene	1006	1002	0.3
3	D‐limonene	1028	1024	3.6
4	γ‐terpinene	1061	1054	0.3
5	Linalool	1104	1095	1.0
6	2‐Decen‐1‐ol	1277	1268	0.3
7	Decanal	1204	1201	0.6
8	Dodecanal	1408	1408	1.0
Sesquiterpene hydrocarbons				
9	δ‐Elemene	1334	1335	0.9
10	α‐Cubebene	1346	1345	0.3
11	α‐Copaene	1378	1374	0.6
12	E‐β‐Bourbonene	1384	1387	1.1
13	β‐Elemene	1395	1389	4.6
14	β‐Caryophyllene	1419	1417	32.5
15	γ‐Elemene	1437	1434	2.1
16	Caryophyllene oxide	1583	1581	4.9
17	E‐β‐farnesene	1454	1454	6.6
18	Alloaromadendrene	1462	1458	1.4
19	Germacrene D	1480	1484	6.9
20	(+)‐Epi‐bicyclosesquiphellandrene	1469	–	0.3
21	Elixene	1391	1391	9.8
22	8‐Isopropenyl‐1,5‐dimethyl‐cyclodeca‐1,5‐diene	1556	–	0.2
23	δ‐Cadinene	1520	1522	6.4
24	Cadina‐1,4‐diene	1531	1533	1.5
25	(*E*)‐Nerolidol	1565	1561	0.8
26	(−)‐Spathulenol	1631	–	0.3
27	Globulol	1590	1590	2.2
28	Ledol	1610	1602	0.3
29	Epiglobulol	1580	–	0.4
Others				
30	Linalyl acetate	1253	–	3.2
31	Decyl isobutyrate	1590	–	0.3
32	Dodecyl isobutyrate	118.4	–	0.6
33	Dodecyl pentanoate	2834	–	2.1
Total identified				97.5
Monoterpene hydrocarbons				7.3
Sesquiterpene hydrocarbons				83.2
Others				6.1

*Note*: –, non identified.

^a^ Retention indices on HP‐5MS column.

^b^ Retention indices according to literature (Adams, 2007).

### 3.2 Biological activity assessment of the essential oil from leaves of *Serevenia buxifolia*

#### 3.2.1 Antioxidant capacity

It is well‐known that the radical scavenging property and antioxidant capacity of herbal plants are beneficial activities attractive for the pharmaceutical industry and food additives manufacturers (Ben Hsouna et al., 2017).

The stable DPPH‐free radical scavenging was used to estimate the potential of the EO and α‐tocopherol. Table 2 shows the radical scavenging activity of *S. buxifolia* EO. The results indicated that the EO of *S. buxifolia* leaves displayed concentration‐dependent antioxidant activity. The radical scavenging activity of the EO significantly increased (*p* < .001), from 29.5% to 66.6% according to the increase in concentration from 100 to 250 μg/mL. The IC_50_ value for DPPH‐scavenging activity of *S. buxifolia* EO was 190.8 μg/mL, which was lower than that of the synthetic antioxidant α‐tocopherol with IC_50_ = 42.6 μg/mL (Table 2). The results showed that the antioxidant activity of *S. buxifolia* EO was moderate compared to the positive control. This is in line with the study of Thirugnanasampandan et al. (2015), who obtained antioxidant activity of *A. monophylla* EO with an IC_50_ value of 198.9 μg/mL. The antioxidant activity of the EO in the present study may be related to the presence of the sesquiterpene hydrocarbon, β‐caryophyllene. A similar result was obtained by Sobrinho et al. (2020), where the EO of *Vernonia chalybaea*, rich in β‐caryophyllene, exhibited considerable antiradical capacity. Meanwhile, other sesquiterpenes, such as elixene, cadinene, limonene, linalool, or terpinene, could also be taken into account. For example, Himed et al. (2019) noted the significant antiradical activity of the limonene‐rich EO of *Citrus limonene*. Monoterpenes (limonene) and sesquiterpenes (caryophyllene) were considered to be responsible for the neutralization of the DPPH radical (Mimica‐Dukic et al., 2004). Many studies have reported that the activity of an EO from plants correlated to the major components and the possible synergistic effects among them (Himed et al., 2019; Sobrinho et al., 2020; Wei & Shibamoto, 2007).

**TABLE 2:** DPPH radical scavenging activity of essential oil of *Serevenia buxifolia* leaves and α‐tocopherol.

Samples	Percentage of DPPH radical scavenging				IC_50_ values (μg/mL)
	100 μg/mL	150 μg/mL	200 μg/mL	250 μg/mL	
Essential oil	5.7^d^ ± 0.03	16.4^c^ ± 0.04	31.9^b^ ± 0.03	55.7^a^ ± 0.08	190.8 ± 1.15
α‐tocopherol	35.3^d^ ± 0.01	47.5^c^ ± 0.05	57.4^b^ ± 0.07	78.7^a^ ± 0.02	42.6 ± 0.14

*Note*: All values are the mean ± SD (*n* = 3). Means within a row with different letters significantly differ by Tukey's test at *p* < .05.

#### 3.2.2 Thrombolytic activity

**FIGURE 2:**
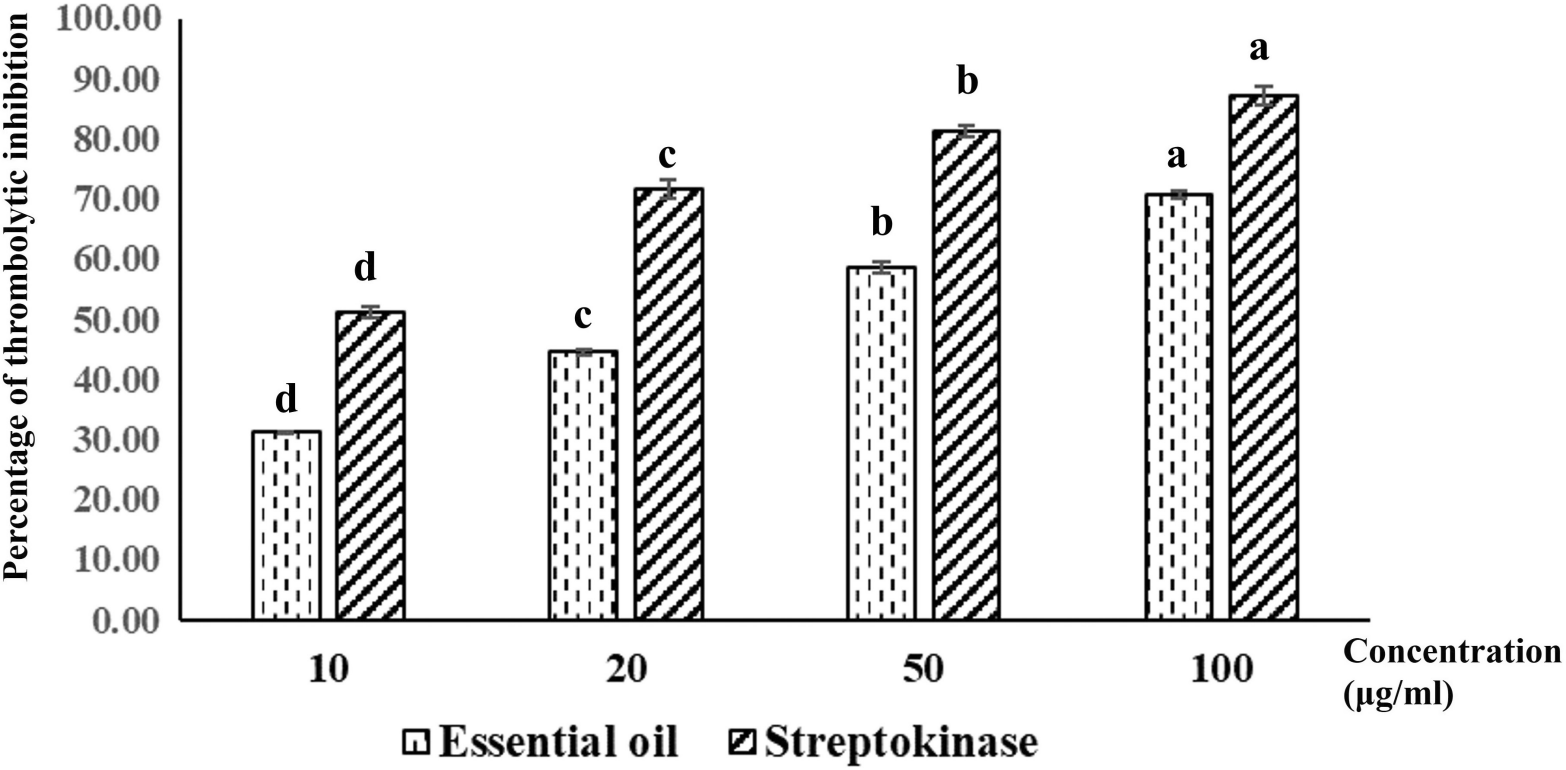
Thrombolytic activity of essential oils from *Serevenia buxifolia* leaves.

Thrombolytic agents, like tissue plasminogen activator (t‐PA), urokinase (UK), and streptokinase (SK) drugs, are used to dissolve blood clots that can obstruct the flow of blood through the circulatory system (Azad et al., 2015; Furie & Furie, 2008). SK can unite and activate the inactive precursor of the enzyme plasmin (plasminogen). It is noted that plasmin is an important proteolytic enzyme that leads to the degradation of fibrin clots (Vinodhini & Kalaiselvi, 2021). However, evidence has indicated that these drugs could cause some side effects. For example, treatment with SK is restricted because of immunogenicity (Jennings, 1996). Thus, the development of recombinant variants of these drugs is necessary (Azad et al., 2015; Farnsworth, 1993; Marder, 1993). Evidence has indicated that herbal plants have an antithrombotic effect preventing thus coronary heart disease and stroke (Azad et al., 2015; Prasad et al., 2007; Ramjan et al., 2014). In the present study, the in vitro thrombolytic potential of the EO of *S. buxifolia* leaves was investigated (Figure 2). The results showed that the *S. buxifolia* leaf oil exhibited slightly lower thrombolytic activity in comparison with the standard, SK. It is noteworthy that the percentage of clot lysis of *S. buxifolia* EO was found to increase in a dose‐dependent manner. The EO of *S. buxifolia* leaves exhibited 70.8% of clot lysis at 100 μg/mL compared with 87.2% clot lysis for the positive standard, SK. On the other hand, the negative control, distilled water, showed a negligible percentage of clot lysis of 8.4%. The mean variation in the percentage of clot lysis between sample (EO), positive, and negative control was found to be statistically significant (*p* < .001). These results coincided with previous studies (Fathima et al., 2015) that studied the thrombolytic activity of EOs from herbal plants. The above‐mentioned results indicate that the EO of *S. buxifolia* leaves contains some plasminogen activators that may support the clot lysis. Our findings were concordant with the study of Vinodhini and Kalaiselvi (2021) who demonstrated the anti‐hemolytic property of *Citrus limetta* oil.

#### 3.2.3 Anti‐hemolytic activity

Increased hemolysis occurs when RBCs are exposed to some toxic factors like hydrogen peroxide (Arfan et al., 2016). Therefore, we assessed whether *S. buxifolia* leaf oil could prevent oxidative damage to the erythrocyte membrane. The results showed that the EO exhibited potent anti‐hemolytic action in a concentration‐dependent way (Table 3). The maximum inhibition of hemolysis was observed at 59.6% RBC membrane stabilization with 100 μg/mL of EO compared to other tested doses, and the IC_50_ value was 65.2 μg/mL. Lysis of erythrocytes was shown to increase with an increase in concentration of EO, that is, %I of hemolytic activity from 27.4% to 59.6% at concentrations from 10 to 100 μg/mL (Table 3). Hence, the above results clearly show that the EO of *S. buxifolia* leaves possesses high anti‐hemolytic activity toward RBCs. *S. buxifolia* leaf oil may exert the protection of erythrocytes from H_2_O_2_‐induced hemolysis. These findings were in line with the studies of Assadpour et al. (2016), who indicated the potential of the EO of *Allium rotundum* in the scavenging of H_2_O_2_ in a concentration‐dependent manner. These authors concluded that the presence of chemical components in the *A. rotundum* EO might lead to electron donation to the hydrogen peroxide.

**TABLE 3:** Antihemolytic activity of essential oil of *Serevenia buxifolia* leaves in addition to IC_50_ values (μg/mL).

Samples	%I of hemolytic activity				IC_50_ values (μg/mL)
	10 μg/mL	20 μg/mL	50 μg/mL	100 μg/mL	
Essential oil	27.4^d^ ± 1.05	32.7^c^ ± 0.04	47.7^b^ ± 0.73	59.6^a^ ± 1.08	65.2 ± 2.15

*Note*: All values are the mean ± SD (*n* = 3). Means within a row with different letters significantly differ by Tukey's test at *p* < .05.

In the present anti‐hemolysis test, the main anti‐hemolytic activity induced by the EO of *S. buxifolia* leaves can be linked to the presence of monoterpenes and sesquiterpenes, as demonstrated by previous studies (Elgendy & Semeih, 2019; Rjeibi et al., 2017; Zouari‐Bouassida et al., 2018). Certain bioactive components are documented as having the potential to generate radicals that attack the erythrocyte membrane, which induces the chain oxidations of proteins and lipids, eventually causing membrane damage due to hemolysis (Vinodhini & Kalaiselvi, 2021).

#### 3.2.4 In vitro anti‐inflammatory activity: inhibition of albumin denaturation

It is known that proteins, namely albumin and trypsin, can activate and release inflammatory mediators from humans and may be involved in inflammation and immunity (Foudah et al., 2021; Ullah et al., 2014). In the present experiment, anti‐denaturation was established using the BSA method to estimate the in vitro anti‐inflammatory property of the EO of *S. buxifolia* leaves. The results are summarized in Table 4. The reference drug, aspirin, also exhibited a concentration‐dependent inhibition of albumin denaturation over the same concentration range. Indeed, the effect of aspirin against BSA heat denaturation was found to be more than 1.1‐fold higher than that of *S.buxifolia* EO. The percentage of protected BSA increased with increasing dose of EO and showed a significant difference (*p* < .001). In particular, we showed a dose‐dependent inhibition of protein (albumin) by *S. buxifolia* leaf oil in the range from 10 to 100 μg/mL. The effect of the reference drug, aspirin, against heat denaturation of the BSA was found to be lower than that of the *S. buxifolia* oil at the same concentration range. These observations were confirmed through IC_50_ values, with 60.2 and 40.3 μg/mL for aspirin and EO, respectively (Table 4). To our best knowledge, the in vitro anti‐inflammatory properties of the EO of *S. buxifolia* leaves have not yet been published. In some of our previous studies, the anti‐inflammatory activity of *S. buxifolia* has been attributed to the presence of alkaloids, terpenoids, and several components (Truong et al., 2019, 2021). Apel et al. (2010) investigated the effects of the EOs of *Myrciaria tenella* and *Calycorectes sellowianus* on both in vitro and in vivo anti‐inflammatory activity and showed the correlation of the main components in the oils (guaiol and β‐caryophyllene) to such activity. Some other studies have also demonstrated the anti‐inflammatory action of the terpenoid‐rich EOs of some herbal plants (de Santana Souza et al., 2014; Del Prado‐Audelo et al., 2021; Gallily et al., 2018; Kim et al., 2020). For instance, Gallily et al. (2018) achieved a difference in the in vitro and in vivo anti‐inflammatory activities of the EOs rich in terpenoids from different *Cannabis* species, which vary according to their composition. Hence, the inflammatory inhibition ability of the EO from *S. buxifolia* in the present study may be attributed to the presence of monoterpenes, sesquiterpenes, and other components. In terms of the inflammation inhibition of β‐caryophyllene, an anti‐inflammatory action is proposed via the inhibition of the main inflammatory mediators, like inducible interlukin‐1 beta (IL‐1 β), interleukin‐6 (IL‐6), nitric oxide synthase (iNOS), tumor necrosis factor‐alfa (TNF‐α), nuclear factor kappa‐light‐chain‐enhancer of activated B cells ((NF‐κB), cyclooxygenase 1 (COX‐1), and cyclooxygenase 2 (COX‐2) Brito et al., 2019; Dahham et al., 2015; Francomano et al., 2019). This could be explained by the fact that the inflammatory action of *S. buxifolia* oil may be due to its main component, β‐caryophyllene, besides the other compounds.

**TABLE 4:** Anti‐inflammatory activity of essential oil of *S. buxifolia* leaves in addition to IC_50_ values (μg/mL).

Samples	Percentage of inhibition				IC_50_ values (μg/mL)
	10 μg/mL	20 μg/mL	50 μg/mL	100 μg/mL	
Essential oil	21.9^d^ ± 0.88	30.3^c^ ± 2.24	41.0^b^ ± 0.75	65.3^a^ ± 1.53	60.2 ± 1.25
Aspirin	34.5^d^ ± 0.95	41.4^c^ ± 1.54	58.6^b^ ± 1.88	73.8^a^ ± 2.34	40.2 ± 0.78

*Note*: All values are the mean ± SD (*n* = 3). Means within a row with different letters significantly differ by Tukey's test at *p* < .05.

#### 3.2.5 In vitro antidiabetic activity

Diabetes is a well‐known serious metabolic disorder which occurs because of the deficiency of insulin secretion or the resistance against insulin. Up to now, significant developments, such as diet therapy, insulin therapy, and pharmacotherapy, are used in the treatment of diabetic patients (Kooti et al., 2016; Mrabti et al., 2018). However, evidence indicates that these treatments still have some side effects, drug resistance (reduction of efficiency), or even toxicity (Dey et al., 2002; Kooti et al., 2015; Mrabti et al., 2018). It is worth noting that, in traditional medicines, a medical plant can be used to treat diabetes without side effects (Kooti et al., 2016). Diabetic patients always need adequate food management to prevent hyperglycemia. It has been documented that inhibition of α‐amylase and α‐glucosidase can lead to slow and prolonged release of glucose into the circulation, thereby retarding glucose release and absorption after food consumption, reducing thus postprandial hyperglycemia (Kumar et al., 2011). Thus, these enzymes play an important role in the management of hyperglycemia‐linked diabetic disease (Aazza et al., 2016; Inthongkaew et al., 2017). The ability of EOs of herbal/aromatic plants to inhibit α‐amylase and α‐glucosidase has been reported (Hichri et al., 2019; Jaradat et al., 2020; Salehi et al., 2020; Siahbalaei et al., 2020). For example, the EO of *Eruca vesicaria* exerted a strong inhibitory activity against α‐amylase and α‐glucosidase, with potencies better than those of the reference drug, acarbose, with IC_50_ values of 0.81 and 0.13 L g/mL, respectively (Hichri et al., 2019). In the present study, the inhibitory potential of *S. buxifolia* EO on α‐amylase and α‐glucosidase was investigated, and the results are represented in Table 5. The effectiveness of the EO on α‐amylase and α‐glucosidase inhibition was compared with that of the treatment drug, acarbose, based on their IC_50_ values. Hichri et al. (2019) indicate that EOs had stronger α‐amylase inhibition than α‐glucosidase. It is noted that high values of IC_50_ show low inhibitory activity. The EO of *S. buxifolia* leaves exhibited considerable antidiabetic activity, with IC_50_ values of 87.8 and 134.9 μg/mL for α‐amylase and α‐glucosidase inhibition, respectively, while the IC_50_ values of the commercial antidiabetic drug, acarbose, were 36.38 and 74.80 μg/mL for the inhibition of α‐amylase and α‐glucosidase, respectively (Table 5). Our results are in contrast to previous studies, which obtained poor inhibition of enzymes related to diabetic disease (Ceylan et al., 2016; Nazir et al., 2021). Recently, Nazir et al. (2021) obtained high inhibitory activity of the EO of the autumn olive plant (*Elaeagnus umbellata*) against α‐amylase and α‐glucosidase (IC_50_ values were 110 and 120 μg/mL, respectively).

**TABLE 5:** The α‐amylase and α‐glucosidase inhibitory activity by acarbose drug and *Serevenia buxifolia* leave oil in addition to their IC_50_ values (μg/mL).

Activities	Concentration (μg/mL)	Percentage of inhibition	
		Essential oil	Acarbose
α‐amylase	50	45.51^d^ ± 1.30	51.23^d^ ± 0.85
	100	50.50^c^ ± 1.25	64.89^c^ ± 0.39
	150	57.58^b^ ± 0.68	69.28^b^ ± 0.97
	200	73.92^a^ ± 1.63	80.84^a^ ± 0.77
	IC_50_ values	87.75 ± 2.35	36.38 ± 1.05
α‐glucosidase	50	29.32^d^ ± 0.76	44.03^d^ ± 1.05
	100	41.02^c^ ± 1.43	55.26^c^ ± 1.00
	150	51.20^b^ ± 0.95	69.57^b^ ± 0.67
	200	68.37^a^ ± 1.11	78.99^a^ ± 0.47
	IC_50_ values	134.89 ± 2.25	74.80 ± 1.23

*Note*: All values are the mean ± SD (*n* = 3). Means within a column with different letters significantly differ by Tukey's test at *p* < .05.

Apparently, the inhibitory ability of *S. buxifolia* EO on these enzymes increased in a dose‐dependent manner. The observed % α‐amylase inhibition potential of the EO samples ranged from 45.51% to 73.92% in the concentration range from 50 to 200 μg/mL; respectively, while the % α‐glucosidase enzyme inhibition potentials ranged from 29.32% to 68.37% (Table 5). Terpenoids have been reported to have antidiabetic effects (Kocharkaur et al., 2019; Kumawat & Kaur, 2020; Nazir et al., 2021). Hence, the achieved antidiabetic potential of leaf EO may be due to the presence of β‐caryophyllene (identified as the main component in the GC/MS analysis of *S. buxifolia* leaf EO; see Table 5), which has been previously documented (Kaur et al., 2018; Kumawat & Kaur, 2020). The D‐limonene (Murali & Saravanan, 2012), germacrene D (Xu et al., 2021), and linalool (More et al., 2014) also possess the capacity to inhibit enzyme‐related diabetic disease.

Our previously shown data on *S. buxifolia* verified its antioxidant and anti‐inflammatory potential (Truong et al., 2019, 2021). In the present study, the EO from *S. buxifolia* leaves was found to be rich in terpenoids, and the most active compounds, such as limonene, linalool, caryophyllene, elixene, and cadinene, which are responsible for the observed in vitro biological activity (i.e., antioxidant, thrombolytic, anti‐hemolytic, anti‐inflammation, and antidiabetic) were nominated based on documentation in the literature (Brito et al., 2019; Elgendy & Semeih, 2019; Francomano et al., 2019; Xu et al., 2021).

## 4 CONCLUSION

In the present study, the chemical composition of the EO extracted from *S. buxifolia* leaves was determined. Thirty‐three components were identified using GC/MS analysis. As a valuable source for traditional medicine, the *S. buxifolia* EO demonstrated in vitro antioxidant, thrombolytic, anti‐hemolysis, anti‐inflammatory, and antidiabetic activities. Thus, the EO of this plant could be considered as an alternative food supplement to treat oxidative stress and stress‐related diseases. Nevertheless, this study was limited to an in vitro evaluation of the EO biological activities; therefore, the observed potential and bioavailability aspects should be addressed in animal models in the future.

## AUTHOR CONTRIBUTIONS

**Kim Ngan Nguyen:** Data curation (equal); formal analysis (equal); methodology (equal). **Nhat Tan Nguyen:** Methodology (equal); validation (equal); writing – original draft (equal). **Khanh Duy Huynh:** Data curation (equal); methodology (equal); resources (equal). **Tomas Ruml:** Writing – review and editing (equal). **Dieu‐Hien Truong:** Conceptualization (equal); data curation (equal); investigation (equal); methodology (equal); project administration (equal); resources (equal); software (equal); supervision (equal); validation (equal); writing – original draft (equal); writing – review and editing (equal).

## CONFLICT OF INTEREST STATEMENT

We, the authors, declare that we have no conflict of interests.

## ETHICS STATEMENT

Ethical approval is not applicable to this study.

## Data Availability

Data are available by contacting the corresponding author by email.
